# Fasting Upregulates PPAR*α* Target Genes in Brain and Influences Pituitary Hormone Expression in a PPAR*α* Dependent Manner

**DOI:** 10.1155/2009/801609

**Published:** 2009-11-18

**Authors:** Bettina König, Christine Rauer, Susann Rosenbaum, Corinna Brandsch, Klaus Eder, Gabriele I. Stangl

**Affiliations:** ^1^Institute of Agricultural and Nutritional Sciences, Martin-Luther-University Halle-Wittenberg, Halle (Saale), Germany; ^2^Chair of Animal Nutrition, Center of Life and Food Sciences Weihenstephan, Technical University Munich, Freising, Germany

## Abstract

PPAR*α* is a lipid-activable transcription factor that mediates the adaptive response to fasting. Recent data indicate an important role of brain PPAR*α* in physiological functions. However, it has not yet been shown whether PPAR*α* in brain can be activated in the fasting state. Here we demonstrate that fasting of rats increased mRNA concentrations of typical PPAR*α* target genes implicated in *β*-oxidation of fatty acids (acyl-CoA oxidase, carnitine palmitoyltransferase-1, medium chain acyl-CoA dehydrogenase) and ketogenesis (mitochondrial 3-hydroxy-3-methylglutaryl-CoA synthase) in pituitary gland and partially also in frontal cortex and diencephalon compared to nonfasted animals. These data strongly indicate that fasting activates PPAR*α* in brain and pituitary gland. Furthermore, pituitary prolactin and luteinizing hormone-*β* mRNA concentrations were increased upon fasting in wild-type mice but not in mice lacking PPAR*α*. For proopiomelanocortin and thyrotropin-*β*, genotype-specific differences in pituitary mRNA concentrations were observed. Thus, PPAR*α* seems to be involved in transcriptional regulation of pituitary hormones.

## 1. Introduction

The ability of animals to survive during food deprivation requires biochemical and physiological responses to the lack of food. The overall metabolic response to fasting operates at numerous levels, and peroxisome proliferator-activated receptor (PPAR)-*α* which belongs to the family of nuclear hormone receptors mediates an adaptive response to fasting. PPAR*α* is directly activated by nonesterified fatty acids (NEFAs) [1, 2] which are liberated from adipose tissue into plasma by lipolytic stimuli and acts as a nutritional state sensor by stimulating the transcription of genes involved in fatty acid uptake through membranes, fatty acid binding in cells, peroxisomal and mitochondrial fatty acid oxidation, ketone body synthesis as well as glycogenolysis and gluconeogenesis [3]. All of these metabolic responses are crucial to shift the body fuel utilization from carbohydrates and fat in the fed state to almost exclusively fat and ketone bodies during fasting and to maintain blood glucose levels to provide tissues such as brain with sufficient amounts of glucose.

PPAR*α* is expressed mostly in tissues with high rates of fatty acid oxidation and peroxisomal metabolism like brown adipose tissue, liver, kidney, and heart [3] but is also expressed in different regions of the brain [4]. Several studies exist that indicate an important role of brain PPAR*α* in physiological functions like neuroprotection [5] and the control of whole-body glucose homeostasis during fasting [6]. Furthermore, specific upregulation of the prolactin gene in a pituitary cell line by PPAR*α* was shown [7] indicating a possible role of PPAR*α* in transcriptional regulation of pituitary hormone production.

Until now it is not known whether PPAR*α* in brain is indeed activated during fasting. It was shown that administration of the PPAR*α* agonist ciprofibrate upregulates typical PPAR*α* target genes such as mitochondrial 3-hydroxy-3-methylglutaryl-(mHMG)-CoA synthase and, to a lesser extent, acyl-CoA oxidase (ACO) and medium chain acyl-CoA dehydrogenase (MCAD; [8]) and increases the rate of fatty acid oxidation in brain homogenates [9].

Thus, this study was designed to test whether fasting would result in an activation of PPAR*α* in brain of rats. PPAR*α* activation was measured by determination of mRNA concentrations of the typical PPAR*α* target genes such as ACO, carnitine palmitoyltransferase (CPT)-1, medium chain acyl-CoA dehydrogenase (MCAD), and mHMG-CoA synthase. A clofibrate-treated group was included to compare the effects of fasting with those of PPAR*α* activation by a synthetic ligand. For analysis, we choose frontal cortex, part of the diencephalon, and the pituitary gland which are known to express PPAR*α* in detectable amounts [4]. We could show a significant upregulation of PPAR*α* target genes particularly in pituitary gland of rats. Considering that and the described regulation of prolactin gene by PPAR*α* in a pituitary cell line [7], we analysed a possible role of brain PPAR*α* in regulation of pituitary hormones during fasting. For that, we analysed mRNA concentrations of prolactin, proopiomelanocortin (POMC), luteinizing hormone (LH)-*β*, follicle-stimulating hormone (FSH)-*β*, growth hormone (GH) and thyrotropin (TSH)-*β* in pituitary gland of fasted and fed PPAR*α* knockout and corresponding wild-type mice. Obtained data indicate an involvement of PPAR*α* in transcriptional regulation of pituitary hormones.

## 2. Materials and Methods

### 2.1. Animal Experiments

Animals were kept individually in Macrolon cages in a room controlled for temperature (22 ± 2°C), relative humidity (50–60%), and light (12 hours light/dark cycle). All experimental procedures described followed established guidelines for the care and handling of laboratory animals and were approved by the council of Saxony-Anhalt. Male Sprague-Dawley rats, with an average initial body weight of 258 g (±17; SD), were randomly assigned to three groups (*n* = 9). All rats were fed a commercial standard basal diet (“altromin 1324,” Altromin GmbH, Lage, Germany). To standardize food intake, the diets were fed daily in restricted amounts of 22 g per day. Water was available ad libitum from nipple drinkers during the whole experiment. The animals were treated with 250 mg/kg of clofibrate in 1 mL sunflower oil (clofibrate group) or with an equal volume of the vehicle sunflower oil (control group and fasting group) by gavage once a day 2 hours after beginning of the light cycle. Animals of the fasting group were fasted 36 hours before killing. During food deprivation, they obtained water instead of sunflower oil by gavage. At day 4 of treatment, animals received the last dose of clofibrate, sunflower oil, and water, respectively, and 7 g of the diet (except fasting group) and were killed 4 hours later by decapitation under light anaesthesia with diethyl ether. Blood was collected into heparinized polyethylene tubes. The brains were removed and dissected. For analysis, the pituitary gland, frontal cortex, and the ventral part of diencephalon were used. The liver was excised. Brain and liver samples for RNA isolation were snap-frozen in liquid nitrogen and stored at −80°C.

Female PPAR*α* knockout mice (129S4/SvJae-*P p a r a* ^tm1Gonz^/J) and corresponding wild-type control mice (129S1/SvImJ) were purchased from the Jackson Laboratory (Bar Harbor, USA). Mice from both genotypes with an average initial body weight of 27.4 g (±1.4; SD) were randomly assigned to two groups. Mice of the control groups (wild-type mice *n* = 10 and PPAR*α* knockout ^**^mice *n* = 10) were fed ad libitum with a commercial standard basal diet (altromin 1324). Mice of the fasting groups (wild-type mice *n* = 10 and PPAR*α* knockout mice *n* = 10) were fasted 48 hours before killing. Mice were killed by decapitation under light anaesthesia with diethyl ether. For RNA analysis, the liver and pituitary gland were excised, snap-frozen in liquid nitrogen, and stored at −80°C.

### 2.2. RT-PCR Analysis

Total RNA isolation from tissues and cDNA synthesis were carried out as described [10]. For RNA isolation from pituitary glands of mice, tissue samples of two animals were pooled. The mRNA expression of genes was measured by real-time detection PCR using SYBR Green I and the Rotor Gene 2000 system (Corbett Research, Mortlake, Australia) as described in [11]. Annealing temperature for all primer pairs (Operon Biotechnologies, Cologne, Germany; Table 1) was 60°C (exception: mouse mHMG-CoA synthase, TSH*β*, 58°C; mouse *β*-actin, 66°C). For determination of mRNA concentration a threshold cycle (C_t_) and amplification efficiency were obtained from each amplification curve using the software Rotor Gene 4.6 (Corbett Research). Calculation of the relative mRNA concentration was made using the amplification efficiencies and the C_t_ values [12]. The housekeeping gene *β*-actin was used for normalization.

### 2.3. Concentration of NEFA

The concentration of NEFA in plasma was determined using an enzymatic reagent kit (Wako Chemicals GmbH, Neuss, Germany).

### 2.4. Statistics

Data of all experiments were analyzed using the Minitab Statistical Software (Minitab, State College, PA, USA). Treatment effects of rat experiment were evaluated by one-way ANOVA. For significant F values (*P* < .05), means of the treatments (fasting, clofibrate) were compared pairwise with the control group by Student's *t*-test. Treatment effects of mice experiment were analyzed by two-way ANOVA with classification factors being treatment (fasting), genotype and the interaction of treatment (fasting) and genotype. In all experiments, means were considered significantly different for *P* < .05.

## 3. Results

### 3.1. Final Body Weights of Rats and Mice

Final body weight of rats was significantly influenced by fasting but not by clofibrate treatment (control: 265 ± 18 g; fasting: 241 ± 12 g; clofibrate treatment: 266 ± 17 g; mean ± SD, *n* = 9). Final body weights of fasted rats were lower than those of nonfasted (control) rats (*P* < .05). Final body weights of mice were significantly influenced by fasting and the genotype (wild-type control, 26.4 ± 1.4 g; wild-type fasting, 23.5 ± 1.5 g; PPAR*α*-knockout control, 27.8 ± 1.4 g; PPAR*α*-knockout fasting, 24.4 ± 1.4 g; mean ± SD; *n* = 10). Final body weights of fasted mice were lower than those of non-fasted mice (*P* < .05); final body weights of PPAR*α*-knockout mice were higher than those of wild-type mice (*P* < .05). The interaction of fasting and genotype had no effect on final body weights.

### 3.2. Concentrations of NEFA in Plasma of Rats

Fasted rats had higher concentrations of NEFA in plasma than control rats whereas those of clofibrate treated rats were unchanged (control: 234 ± 37 *μ*mol/L; fasted: 676 ± 162; clofibrate: 216 ± 32 *μ*mol/L; *n* = 9 for each group; *P* < .05).

### 3.3. mRNA Concentrations in Rat Brain and Pituitary Gland

**Figure 1:**
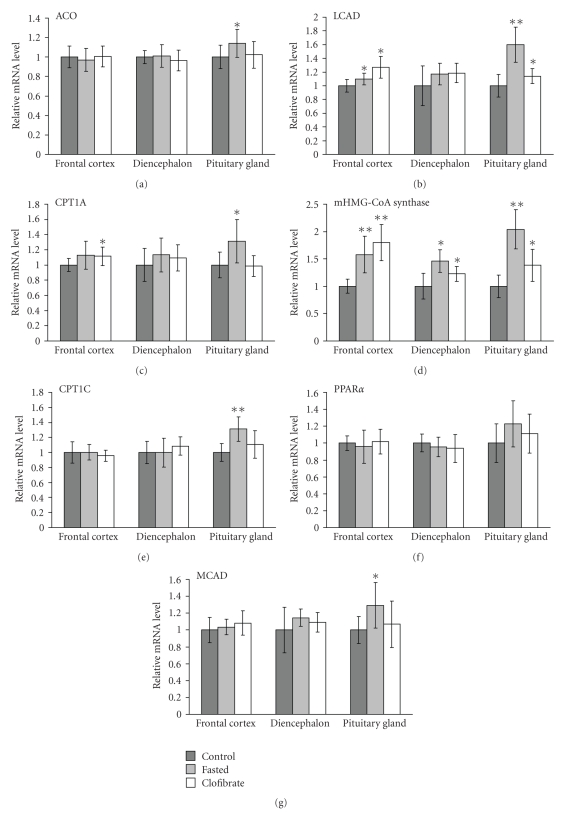
Effect of fasting or clofibrate treatment on mRNA concentration of acyl-CoA oxidase (ACO), carnitine palmitoyltransferases (CPT)-1A and -1C, medium chain acyl-CoA dehydrogenase (MCAD), long chain acyl-CoA dehydrogenase (LCAD), mitochondrial 3-hydroxy-3-methylglutaryl-(mHMG)-CoA synthase, and peroxisome proliferator-activated receptor (PPAR)-*α* in different brain areas of rats. All rats obtained a standard basal diet for 4 days. Animals of the fasting group were fasted 36 hours before killing. Rats of the clofibrate group were treated orally with 250 mg/kg of clofibrate per day. Values are means ± SD (*n* = 9). Symbols indicate significant difference from control rats (*P < .05, **P < .001).

To elucidate a possible activation of PPAR*α* in rat brain by fasting, we measured mRNA concentrations of typical PPAR*α* target genes in frontal cortex, diencephalon, and pituitary gland of fasted and non-fasted rats. mRNA concentration of ACO was higher in pituitary gland of fasted rats compared to control rats (*P* < .05; see Figure 1). Oral treatment of a third group of rats with the synthetic PPAR*α* agonist clofibrate did not change mRNA concentration of ACO in all brain areas examined and pituitary gland compared to control rats (Figure 1).

Concentration of CPT1A mRNA, which represents the widely expressed liver isoform of CPT1 [13], was higher in pituitary gland of fasted rats compared to control rats (*P* < .05; see Figure 1). In clofibrate treated rats, CPT1A mRNA concentration was higher in frontal cortex compared to control rats (*P* < .05; Figure 1). mRNA concentration of CPT1C, the brain specific isoform of CPT1 [14], was higher in pituitary gland of fasted rats compared to control rats (*P* < .05; Figure 1). Clofibrate treatment did not change mRNA concentration of CPT1C in all brain areas examined and pituitary gland compared to control rats (Figure 1). MCAD mRNA concentration was higher in pituitary gland of fasted rats compared to control rats (*P* < .05; Figure 1). Clofibrate treatment did not change mRNA concentration of MCAD in all examined tissues (Figure 1). In fasted and clofibrate treated rats, mRNA concentrations of LCAD were higher in frontal cortex and pituitary gland than those in control rats (*P* < .05; Figure 1).

mHMG-CoA synthase mRNA concentration was higher in frontal cortex, diencephalon, and pituitary gland of fasted and clofibrate treated compared to control rats (*P* < .05; Figure 1).

Fasting tended to increase the mRNA concentration of PPAR*α* in pituitary gland compared to nonfasting (*P* = .073; Figure 1). Clofibrate treatment did not alter mRNA concentration of PPAR*α* in all brain areas examined and pituitary gland compared to control rats (Figure 1).

### 3.4. mRNA Concentrations in Rat Liver

**Figure 2:**
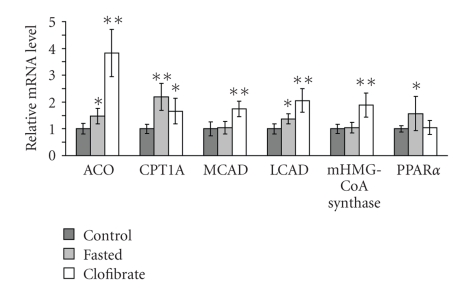
Effect of fasting or clofibrate treatment on mRNA concentration of acyl-CoA oxidase (ACO), carnitine palmitoyltransferases (CPT)-1A, medium chain acyl-CoA dehydrogenase (MCAD), long chain acyl-CoA dehydrogenase (LCAD), mitochondrial 3-hydroxy-3-methylglutaryl-(mHMG)-CoA synthase, and peroxisome proliferator-activated receptor (PPAR)-*α* in the liver of rats. All rats obtained a standard basal diet for 4 days. Animals of the fasting group were fasted 36 hours before killing. Rats of the clofibrate group were treated orally with 250 mg/kg of clofibrate per day. Values are means ± SD (*n * = 9). Symbols indicate significant difference from control rats (*P < .05, **P < .001).

In liver, mRNA concentrations of ACO, CPT1A, LCAD, and PPAR*α* were higher in fasted than in control rats (*P* < .05; Figure 2). In the liver of clofibrate treated rats, mRNA concentrations of ACO, CPT1A, MCAD, LCAD and mHMG-CoA synthase were higher than in control rats (*P* < .05; Figure 2).

### 3.5. mRNA Concentrations in Pituitary Gland of PPAR*α* Knockout and Wild-Type Mice

**Figure 3:**
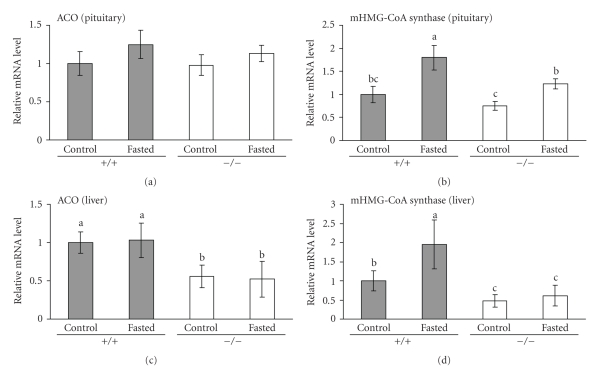
Effect of fasting on mRNA concentrations of acyl-CoA oxidase (ACO) and mitochondrial 3-hydroxy-3-methylglutaryl-(mHMG)-CoA synthase in pituitary gland and liver of wild-type (+/+) and PPAR*α* knockout mice (−/−). Mice of both genotypes were either fasted for 48 hours (control group) or fed a standard rodent diet ad libitum for 48 hours (fed group). Livers and pituitary glands of mice were excised, pituitary glands from two animals were pooled, total RNA was extracted, and mRNA abundances were determined by real-time detection RT-PCR analysis using *β*-actin for normalization. Bars represent means ± SD (liver: *n* = 10; pituitary: *n* = 5). Means without a common letter differ, *P* < .05. Significant effects (*P* < .05) from two-way ANOVA: mHMG-CoA synthase (pituitary): genotype, fasting, fasting × genotype (*P* = .09); ACO (liver): genotype; mHMG-CoA synthase (liver): fasting, genotype, fasting × genotype.

To elucidate a possible role of PPAR*α* in expression of pituitary hormones, wild-type and PPAR*α* knockout mice were fasted for 48 hours. In pituitary gland, the PPAR*α* target gene ACO was not significantly increased in fasted mice compared to non-fasted mice of both genotypes (Figure 3). Fasting led to an increase of mHMG-CoA synthase mRNA concentration in pituitary gland of wild-type mice (*P* < .05; see Figure 3). Fasting did also increase mHMG-CoA synthase mRNA in PPAR*α* knockout mice but this effect was less than that observed in wild-type mice (*P* < .05; Figure 3). For pituitary mHMG-CoA synthase, there was a tendency to interaction of genotype and fasting (*P* = .090). For comparison, mRNA levels of ACO and mHMG-CoA synthase were also measured in liver of mice. Liver ACO mRNA concentration was lower in PPAR*α* knockout than in wild-type mice (*P* < .05) but was not increased upon fasting for 48 hours in both genotypes (Figure 3). Liver mHMG-CoA synthase was increased upon fasting in wild-type mice but not in PPAR*α* knockout mice and was lower in PPAR*α* knockout mice than in wild-type mice (*P* < .05; see Figure 3). There was a significant interaction of genotype and fasting for liver mHMG-CoA synthase mRNA (*P* < .05).

**Figure 4:**
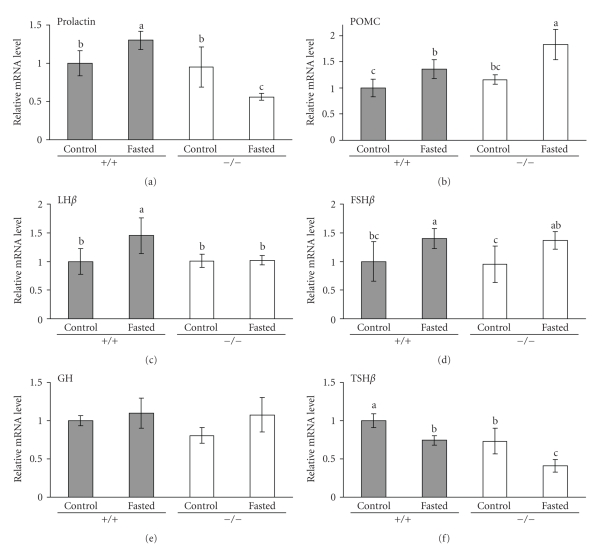
Effect of fasting on mRNA concentrations of prolactin, proopiomelanocortin (POMC), luteinizing hormone (LH)-*β*, follicle-stimulating hormone (FSH)-*β*, growth hormone (GH), and thyrotropin (TSH)-*β* in pituitary gland of wild-type (+/+) and PPAR*α* knockout mice (−/−). Mice of both genotypes were either fasted for 48 hours (control group) or fed a standard rodent diet ad libitum for 48 hours (control group). Pituitary glands of mice were excised, pooled from two animals, total RNA was extracted, and mRNA abundances were determined by real-time detection RT-PCR analysis using *β*-actin for normalization. Bars represent means ± SD (*n* = 5). Means without a common letter differ, *P* < .05. Significant effects (*P* < .05) from two-way ANOVA: prolactin: genotype, fasting × genotype; POMC: fasting, genotype; LH*β*: fasting, fasting × genotype (*P* = .053); FSH*β*: fasting; TSH*β*: fasting, genotype.

Next we analysed mRNA concentrations of prolactin, POMC, LH*β*, FSH*β*, GH, and TSH*β* in pituitary gland of fasted and non-fasted wild-type and PPAR*α* knockout mice. mRNA concentration of prolactin in pituitary gland was higher in fasted than in non-fasted wild-type mice (*P* < .05; see Figure 4). In PPAR*α* knockout mice, fasting caused a decrease of prolactin mRNA concentration (*P* < .05; see Figure 4). Prolactin mRNA concentration of wild-type and PPAR*α* knockout control (fed) mice did not differ. For prolactin mRNA concentration in pituitary gland, there was an interaction of genotype and fasting (*P* < .05).

mRNA concentration of POMC in pituitary gland was higher in fasted than in non-fasted mice of both genotypes (*P* < .05; see Figure 4). POMC mRNA concentration was higher in fasted PPAR*α* knockout mice than in fasted wild-type mice (*P* < .05; see Figure 4). There was no difference in POMC mRNA levels between non-fasted groups of both genotypes.

Fasting led to an increase of mRNA concentration of LH*β* in pituitary gland of wild-type (*P* < .05) mice but not of PPAR*α* knockout mice (Figure 4). For LH*β* mRNA concentration, there was a tendency to interaction of genotype and fasting (*P* = .053). FSH*β* mRNA concentration in pituitary gland was higher in fasted than in non-fasted mice of both genotypes (*P* < .05; see Figure 4). For GH mRNA concentration, no significant differences were observed between all four groups of mice (Figure 4).

Fasting led to a significant decrease of TSH*β* mRNA in pituitary gland of both wild-type and PPAR*α* knockout mice (*P* < .05; Figure 4). Furthermore, TSH*β* mRNA level was lower in PPAR*α* knockout mice than in the corresponding group of wild-type mice (*P* < .05; see Figure 4).

## 4. Discussion

This study was designed to investigate the effect of fasting on the expression of PPAR*α* target genes in rat brain. As expected, food deprivation for 36 hours led to a considerable loss of body weight of the rats. Mobilization of triacylglycerols from adipose tissue by fasting moreover caused an increase in the concentrations of NEFA in plasma whereas clofibrate treatment did not change plasma NEFA concentrations of the rats.

In this study, we could demonstrate for the first time that fasting of rats for 36 hours upregulates mRNA concentration of ACO, CPT1, MCAD, LCAD, and mHMG-CoA synthase in pituitary gland and, partially, also in frontal cortex and diencephalon of the brain. All enzymes are typical PPAR*α* target genes or PPAR*α* responsive genes (LCAD; reviewed in [3]), and the observed upregulation of these genes during fasting strongly indicates PPAR*α* activation in brain during food deprivation. This finding supports the hypothesis of Knauf et al. [6] that PPAR*α* activation in brain is vital during fasting.

It has been shown that PPAR*α* is well expressed in various brain areas and that it is colocalized with ACO indicating that also in brain tissue peroxisomal *β*-oxidation is regulated by PPAR*α* [4, 15]. Although it is generally thought that the liver supplies the brain with ketone bodies as a glucose-replacing fuel, for example, during fasting, it has been demonstrated that fatty acids are oxidized by the brain and isolated astrocytes [9, 16] and that cultured astrocytes are able to synthesize ketone bodies from fatty acids [16] assuming that astrocytes may provide neurons with ketone bodies as a glucose-replacing fuel in vivo. This hypothesis is supported by our findings that genes involved in *β*-oxidation of fatty acids (ACO, CPT1, MCAD, LCAD) and mHMG-CoA synthase which is implicated in ketogenesis are upregulated upon fasting in rat brain and pituitary gland.

Among the PPAR*α* target genes analysed in brain, mHMG-CoA synthase was upregulated strongest by fasting. Regarding the different brain areas tested, upregulation of PPAR*α* responsive genes in general was stronger in pituitary gland than in frontal cortex and diencephalon. mHMG-CoA synthase and LCAD were the only PPAR*α* responsive genes that were considerably upregulated by clofibrate treatment in brain, with the strongest effects in frontal cortex followed by pituitary gland and diencephalon. These distinct relative intensities of upregulation in the three brain areas observed by fasting and clofibrate treatment indicate that the different responses in frontal cortex, diencephalons, and pituitary gland upon fasting are probably not due to different expression levels of PPAR*α*.

In general, the effect of fasting on PPAR*α* responsive genes in brain was much stronger than that of clofibrate treatment. In contrast, in the liver, upregulation of most of these genes was much stronger in clofibrate treated than in fasted rats. This discrepancy may reflect the different access of PPAR*α* agonists to the brain. It has been demonstrated that fibrates can access the brain but that they cross the blood-brain barrier slowly [9, 17]. During fasting, hydrolysis of triacylglycerols in adipose tissue is stimulated leading to increased concentrations of plasma NEFA which then activate PPAR*α* in the liver. We suggest that the observed activation of PPAR*α* in brain by fasting was accomplished by the strong increase in NEFA concentration in plasma of fasted rats. It has been demonstrated that the access of circulating NEFA to the central nervous system is generally proportional to their plasma concentration [18]. Thus, although both, clofibrate, and NEFA are potent PPAR*α* agonists [19], the PPAR*α* activation by NEFA released upon fasting is stronger than that of orally administered clofibrate.

NEFA that are released upon fasting also upregulate PPAR*α* transcription in the liver [20], as could be also observed in rats fasted for 36 hours in this study. In brain, PPAR*α* was slightly but not significantly (*P* = .073) up-regulated only in pituitary gland which corresponds well with the upregulation pattern of its target genes which was also the strongest in this area. Clofibrate treatment of rats did not change mRNA concentration of PPAR*α* which is in agreement with other studies showing that PPAR*α* activation by fibrates does not necessarily upregulate expression of PPAR*α* [21, 22].

In the liver of rats, there was weak or even no upregulation of PPAR*α* responsive genes after 36 hours of fasting. This is consistent with data showing that mRNA concentrations of PPAR*α* target genes in the liver of rats are upregulated after 24 hours of fasting, but decrease after longer fasting times to control levels [23]. In a preliminary experiment, we analysed expression of PPAR*α* target genes in brain of rats fasted for 24 hours. Interestingly, upregulation of PPAR*α* target genes in brain after 24 hours of fasting was much weaker than after 36 hours of fasting (data not shown) indicating that activation of PPAR*α* in brain is delayed compared to that in liver. Further experiments regarding this time dependency of PPAR*α* activation in brain and liver may be helpful to explain this phenomenon.

As already mentioned, the prolactin gene is activated by PPAR*α* as demonstrated in a rat pituitary tumor cell line and in reporter gene assays [7]. Thus, our results with fasted rats prompted us to investigate whether PPAR*α* activation upon fasting is involved in regulation of hormone production in pituitary gland. Fasting led to an upregulation of mHMG-CoA synthase in pituitary gland of wild-type mice indicating PPAR*α* activation. However, also in PPAR*α* knockout mice mRNA concentration of mHMG-CoA synthase was increased upon fasting but not as strong as in wild-type mice. In contrast, the upregulation of mHMG-CoA synthase in livers of wild-type mice upon fasting could not be observed in the livers of PPAR*α* knockout mice indicating the PPAR*α*-dependent regulation of mHMG-CoA synthase in liver. Thus, additional mechanisms seem to be involved in upregulation of mHMG-CoA synthase mRNA in pituitary gland upon fasting. It has been shown that in skeletal muscle PPAR*β*/*δ* can compensate the lack of PPAR*α* in regulation of fatty acid homeostasis during starvation [24]. Since PPAR*β*/*δ* is also ubiquitously expressed in brain [4] it is possible that it can compensate at least in part the lack of PPAR*α* mediated upregulation of mHMG-CoA synthase in brain of PPAR*α* knockout mice upon fasting. The fact that no upregulation of ACO was observed in liver and pituitary gland of mice fasted for 48 hours is consistent with the marginal upregulation of ACO observed in rats after 36 hours of fasting and is attributed to the increased fasting time [23].

Analysis of mRNA concentrations of several genes of pituitary hormones in wild-type and PPAR*α* knockout mice revealed that prolactin gene mRNA was upregulated 1.3-fold in wild-type mice upon fasting but was downregulated about 50% in fasted PPAR*α* knockout mice. Thus, our data demonstrate for the first time that the prolactin gene is transcriptionally regulated by PPAR*α* in vivo and support the findings of Tolon et al. [7]. They demonstrated that the rat prolactin promoter is stimulated by PPAR*α* by a mechanism different from that commonly described for PPAR*α* target genes involving interaction of the PPAR*α*/RXR*α* heterodimer complex with a PPRE [7].

Besides prolactin, also for the LH*β* mRNA a genotype-dependent response to fasting was observed. The 1.5-fold up-reguation of LH*β* mRNA concentration by fasting in wild-type mice was not observed in PPAR*α* knockout mice (*P* value for interaction of treatment (fasting) and genotype: .053). This indicates that PPAR*α* may also be involved in regulation of LH*β* expression in pituitary gland during fasting. Fasting also led to about 1.4-fold increases in pituitary FSH*β* mRNA concentration; however this effect was observed both in wild-type and in PPAR*α* knockout mice indicating that PPAR*α* is not involved in regulation of FSH*β* mRNA during fasting in mice.

POMC mRNA concentration was higher in pituitary gland of PPAR*α* knockout than of wild-type mice and it was induced in both genotypes upon fasting. POMC, a multifunctional precursor protein of a number of bioactive peptides, is produced both in pituitary gland and hypothalamus. Interestingly, recently it was described that in the fed state hypothalamic POMC mRNA concentration is also higher in PPAR*α* knockout than in wild-type mice; however it was downregulated upon 24 hours of fasting in both genotypes [6]. This different behaviour of POMC mRNA upon fasting may result from the fact that POMC expression in hypothalamus and pituitary is controlled by independent sets of enhancers [25].

During fasting, mRNA concentration of TSH*β* was reduced in pituitary gland of both wild-type and PPAR*α* knockout mice. Downregulation of TSH*β* is part of a series of changes in the hypothalamic-pituitary-thyroid axis [26]. Interestingly, TSH*β* mRNA concentration was lower in mice lacking PPAR*α* than in wild-type mice indicating a role for PPAR*α* in regulation of TSH*β* that should be analysed in future studies.

## 5. Conclusions

In conclusion, the data of the present study show for the first time that fasting of rats upregulates typical PPAR*α* target genes in frontal cortex, diencephalon, and pituitary gland. This strongly indicates that free fatty acids released upon energy restriction from adipose tissue activate PPAR*α* not only in liver but also in brain. Besides a possible role of brain PPAR*α* in regulation of ketone body synthesis it seems to be also involved in control of pituitary hormone production. Thus, regarding the multitude of genes regulated by PPAR*α*, not only PPAR*α* activation in liver, but also activation of PPAR*α* in brain seems to be an important step in adoption to fasting.

## Figures and Tables

**Table 1:** Characteristics of the specific primers used for RT-PCR analysis.

Gene	Forward primer (5′ to 3′)	Reverse primer (5′ to 3′)	PCR product size (bp)	NCBI GenBank
*β*-Actin (rat)	ATCGTGCGTGACATTAAAGAGAAG	GGACAGTGAGGCCAGGATAGAG	429	BC063166
ACO (rat)	CTTTCTTGCTTGCCTTCCTTCTCC	GCCGTTTCACCGCCTCGTA	415	NM017340
CPT1A (rat)	GGAGACAGACACCATCCAACATA	AGGTGATGGACTTGTCAAACC	416	NM031559
CPT1C (rat)	CATCTCCAGCAAGCAATCAA	GATCCCCAATACCCCTGTCT	299	BC105882
LCAD (rat)	AAGGATTTATTAAGGGCAAGAAGC	GGAAGCGGAGGCGGAGTC	380	NM012849
MCAD (rat)	CAAGAGAGCCTGGGAACTTG	CCCCAAAGAATTTGCTTCAA	154	NM016986
Mitochondrial HMG-CoA synthase (rat)	GGCCTTGGACCGATGCTATGC	GGGAGGCCTTGGTTTTCTTGTTG	323	BC083543
PPAR*α* (rat)	CCCTCTCTCCAGCTTCCAGCCC	CCACAAGCGTCTTCTCAGCCATG	555	NM013196
*β*-Actin (mouse)	ACGGCCAGGTCATCACTATTG	CACAGGATTCCATACCCAAGAAG	87	NM007393
ACO (mouse)	CAGGAAGAGCAAGGAAGTGG	CCTTTCTGGCTGATCCCATA	189	NM015729
LH*β* (mouse)	GTCCCAGGACTCAACCAATG	GGGAGGGAGGGATGATTAGA	110	NM008497
Mitochondrial HMG-CoA synthase (mouse)	CCTCTGTGAATCCTGGGTGT	CTGTGGGGAAAGATCTGCAT	141	NM008256
POMC (mouse)	GGGTCCCTCCAATCTTGTTT	GCACCAGCTCCACACATCTA	137	NM008895
Prolactin (mouse)	CTCAGGCCATCTTGGAGAAG	TCGGAGAGAAGTCTGGCAGT	174	NM011164
FSH*β* (mouse)	AGGGAGGAAAGGAAAGTGGA	AGCCAGCTTCATCAGCATTT	202	NM008045
GH (mouse)	ACGCGCTGCTCAAAAACTAT	GCTAGAAGGCACAGCTGCTT	120	NM008117
TSH*β* (mouse)	TCAACACCACCATCTGTGCT	TCTGACAGCCTCGTGTATGC	239	NM009432
